# A mechanism for revising accreditation standards: a study of the process, resources required and evaluation outcomes

**DOI:** 10.1186/s12913-014-0571-8

**Published:** 2014-11-21

**Authors:** David Greenfield, Mike Civil, Andrew Donnison, Anne Hogden, Reece Hinchcliff, Johanna Westbrook, Jeffrey Braithwaite

**Affiliations:** Centre for Clinical Governance Research, Australian Institute of Health Innovation, Faculty of Medicine, University of New South Wales, Sydney, NSW 2052 Australia; Chair National Standing Committee - Standards for General Practice, The Royal Australian College of General Practitioners, Melbourne, Australia; Centre for Health Systems and Patient Safety Research, Australian Institute of Health Innovation, University of New South Wales, Paddington, NSW Australia

**Keywords:** Accreditation, Healthcare, Standards, General practice, Research

## Abstract

**Background:**

The study objective was to identify and describe the process, resources and expertise required for the revision of accreditation standards, and report outcomes arising from such activities.

**Methods:**

Secondary document analysis of materials from an accreditation standards development agency. The Royal Australian College of General Practitioners’ (RACGP) documents, minutes and reports related to the revision of the accreditation standards were examined.

**Results:**

The RACGP revision of the accreditation standards was conducted over a 12 month period and comprised six phases with multiple tasks, including: review methodology planning; review of the evidence base and each standard; new material development; constructing field trial methodology; drafting, trialling and refining new standards; and production of new standards. Over 100 individuals participated, with an additional 30 providing periodic input and feedback. Participants were drawn from healthcare professional associations, primary healthcare services, accreditation agencies, government agencies and public health organisations. Their expertise spanned: project management; standards development and writing; primary healthcare practice; quality and safety improvement methodologies; accreditation implementation and surveying; and research. The review and development process was shaped by five issues: project expectations; resource and time requirements; a collaborative approach; stakeholder engagement; and the product produced. The RACGP evaluation was that participants were positive about their experience, the standards produced and considered them relevant for the sector.

**Conclusions:**

The revision of accreditation standards requires considerable resources and expertise, drawn from a broad range of stakeholders. Collaborative, inclusive processes that engage key stakeholders helps promote greater industry acceptance of the standards.

## Background

Government, quality improvement and accreditation agencies frequently engage in the development or revision of clinical and organisational standards. These are significant tasks that utilise considerable human and financial resources [1]. Different organisations produce standards according to their own processes and requirements, and it is believed that inclusive processes result in greater acceptance of the standards produced [1]. However, we do not know what might be evidence-informed practice in the development or revision of accreditation standards [2]. To date, no empirical study has been published that sought to identify the process, resources and expertise required for either of these endeavours [2]. This is a significant gap in the evidence base for the healthcare accreditation field [2-4], as accreditation programs have increasingly become an important strategy by which governments seek to regulate healthcare quality and safety [5,6]. There are now more than 44 health service accreditation programs which have been implemented in over 70 countries [3,7].

The aim of this study was to identify and describe the process, resources and expertise required for, and to report evaluation outcomes from, a revision of a set of healthcare accreditation standards. The standards and associated revision activities under examination concern the *Standards for General Practice (4th Edition)* developed and revised by The Royal Australian College of General Practitioners (RACGP), which are used for accrediting general practice nationally. Through implementing an instrumental case study [8,9] of the RACGP accreditation standards we sought to highlight the process, resource and expertise issues relevant for other accreditation standard setting bodies. The RACGP is representative of other bodies that similarly have responsibility for the development and revision of standards, but do not themselves apply or assess services using the standards [10]. Previous studies have revealed the common issues and challenges facing standard setting and accreditation bodies [7,11].

## Methods

### Study context

The Accreditation Collaborative for the Conduct of Research, Evaluation and Designated Investigations through Teamwork (ACCREDIT) was funded across 2011–15 to investigate health service accreditation in Australia [12]. The collaboration comprises university researchers, accreditation agency personnel and staff from leading quality improvement bodies in Australia (Table 1). The collaboration was awarded an Australian Research Council Linkage Project grant (LP100200586) in 2010. Ethics approval for the study was given by the University of New South Wales (UNSW) Human Research Ethics Committee (approval number HREC 10274). The ACCREDIT study protocols are publically available [12-15] and are informed by previous accreditation research conducted by UNSW and The Australian Council on Healthcare Standards (ACHS) [10-12,16-24], including reviews of the healthcare accreditation literature [2-4].

**Table 1:** **ACCREDIT collaborative partners**

**Partner category**	**Organisations**
University researchers	• Centre for Clinical Governance Research, and Centre for Health Systems and Safety Research, in the Australian Institute of Health Innovation at The University of New South Wales
Accreditation agencies	• Australian General Practice Accreditation Limited
	• Aged Care Standards and Accreditation Agency
	• The Australian Council on Healthcare Standards
Quality improvement bodies	• Australian Commission on Safety and Quality in Health Care
	• New South Wales Clinical Excellence Commission

### Setting

Australia has over 7,100 general practices in which more than 23,500 doctors work. There were 125 million consultation services provided during 2010–11, costed at A$5.3 billion through the Medicare Benefits Scheme [25] (this excludes out of pocket expenses of patients). In 2011–12 there were over 4000 general practices accredited against the RACGP *Standards for General Practice* [26]. The standards cover five areas: practice services (7 standards and 19 criteria); rights and needs of patients (1 standard and 3 criteria); safety, quality improvement and education (2 standards and 7 criteria); practice management (2 standards and 4 criteria); and physical factors (3 standards and 8 criteria). (See: http://www.racgp.org.au/your-practice/standards/standards4thedition/).

### Study methodology

An expert group was formed by UNSW researchers and RACGP staff. During 2012, they collaboratively conducted a study with three stages. First, informed by the accreditation and evaluation literatures, the expert group purpose-designed an analysis framework with seven categories including: phase, task, objective, time frame, components, people involved and National Expert Committee – Standards for General Practice (NEC-SGP) involvement. The role of the NEC-SGP includes developing and maintaining standards for general practices, and ensuring that the standards reflect quality practice and are independent of government policies and initiatives. The NEC-SGP comprises experts in standards development with professional backgrounds including general practitioners, practice nurses and managers, and a consumer representative. Since 2011 the NEC-SGP is known as the National Standing Committee – Standards for General Practice. Second, using the framework, thematic analysis [8] of RACGP documents, minutes and evaluation reports related to the revision of the accreditation standards was conducted. More than 50 documents were accessed from the RACGP information system. Third, the group reviewed the findings to clarify the process, resources and expertise utilised, and reported evaluation outcomes. Over several months the expert group discussed the findings in meetings and electronic forums to work through the material, with differences resolved by negotiation [8].

## Results

The analysis framework facilitated the identification of a standards review process comprising six sequential and overlapping phases with multiple components (Table 2). The six phases occurred over a 12 month period across 2009–10. Phase 1 comprised the ‘review methodology planning’ phase, which occurred over two months. This phase involved two tasks: developing the review feedback methodology and tools; and reviewing the evidence base for information on methods of standards development. The review of the evidence base and current standards were the tasks that formed Phase 2, which was completed over a five month period. Following Phase 1, and overlapping with Phase 2, Phase 3 was a five month activity requiring the development of new material for the new standards. The construction of field trial methodology, the sole task in Phase 4, occurred in parallel and was completed in two months. The completion of the initial four phases led to Phase 5 and the combined task of drafting, trailing and refining the new standards, which occupied five months. To complete the project, the final task was the formatting and production of the new standards. This was Phase 6 and occurred over a two month period.

**Table 2:** **Analysis of the review process for the RACGP** ***Standards for general practices (4th edition)***

**Phase**	**Task**	**Objective(s)**	**Time frame**	**Components**	**People involved**	**National Expert Committee – Standards for General Practice (NEC-SGP) involvement**
1 Development of review methodology and tools	Develop the review feedback methodology and tools	Develop methodology to collect feedback from members and stakeholders on current standards	1/09/09 – 30/10/09	Review methodology of previous standards review	Senior Project Officer	Review documentation and meet
		Develop method of collection and analysis of feedback		Review and update draft tools	Secretariat	Decide on accepted review methodology (October)
				Recommend to NEC-SGP review methodology	Project Manager	Recommend review methodology to RACGP Council (November)
					NEC-SGP (5)	RACGP Council ratify review methodology
	Review the evidence base for current method of standards development	Ensure development of the new standards are supported by latest evidence	1/09/09 – 30/10/09	Literature search of methods of standards development and assessment	Senior Project Officer	Secretariat provide recommendations to NEC-SGP on methodology of standards development (October meeting)
				Recommendations to NEC-SGP on how to develop and assess standards	Secretariat	NEC-SGP reviews evidence and accept recommendations on methodology of standards development (October meeting)
				Council acceptance of methodology, timeline and cost	Project Manager	NEC-SGP recommends methodology, timeline and budget required to RACGP Council
					NEC-SGP (5)	RACGP Council ratifies methodology of standards development process and approves budget (November meeting)
2 Reviewing the evidence base and the current standards	Review the evidence base for current standards	Ensure new edition includes material that is supported by latest evidence	1/09/09 – 31/12/09	Literature search of each criteria in current edition	Senior Project Officer	NEC-SGP decide on inclusion or exclusion of current criteria based on evidence presented (January 2010 meeting)
				Recommendations to NEC-SGP on latest trends relating to each criteria’s relevance to the next edition	Secretariat	Decide on membership of subcommittees and chairs; methodology and tools; new material (e.g. e-health, governance, IC, present material)
					NEC-SGP (5)	
	Review the current standards	All feedback on current edition is considered	1/10/09 - 31/01/10	Review feedback collected by RACGP since release of current edition	Project Manager	Secretariat provides analysis of feedback to subcommittees
		New material for possible inclusion in revised edition is identified		Collection of feedback from members and stakeholders via focus groups, online surveys and written submissions	Secretariat	
				Analysis of feedback		
				Recommendations to NEC-SGP and subcommittees on material identified from formal feedback		
3 New material development	Develop new material for new standards	Material included in new edition is evidence based	1/11/09 – 31/03/10	New material is matched with evidence - literature search relating to new material	Senior Project Officer	Secretariat draft new material
				New criteria, indicators and explanatory material are drafted	Senior Project Officer	Sub committees recommend changes to drafts, approve draft of new material and make recommendations to NEC-SGP
					Project Manager	NEC-SGP accept recommendations of sub committees
					Senior Project Officer	NEC-SGP approve draft of new material for trial
					Working groups	NEC-SGP recommend draft to RACGP Council for endorsement for trial
					Secretariat	
4 Develop field trial methodology	Develop field trial methodology	Prepare field trial of revised standards	1/1/10 – 28/2/10	Methodology of field trial of new material is described	Senior Project Officer	Secretariat design trial methodology and tools
				Trial tools are developed	GP research expert	Subcommittee approve to methodology and tools
					Secretariat	Subcommittees recommend methodology and tools to NEC-SGP
					NEC-SGP (5)	NEC-SGP approve trial methodology (April meeting)
						NEC-SGP recommend methodology and tools to RACGP Council (April meeting)
5 Drafting, trials and feedback about the new standards	Seek feedback, trial and refine draft new standards	Ensuring new edition of standards meet needs of RACGP and stakeholders	1/4/10 – 31/8/10	First line feedback groups provide an early indication of the feasibility and acceptability of proposed explanatory material and indicators to the working parties	Accreditation agencies (participant numbers not specified)	Subcommittees approve changes and recommend penultimate version to NEC-SGP
				On line survey for participation by all interested general practice professionals	Project Manager	NEC-SGP approves penultimate draft (July meeting)
				Written submissions	Senior Project Officer	NEC-SGP recommends final Standards to RACGP Council for endorsement
				Focus groups	Secretariat	RACGP Council endorses Standards (August meeting)
				Trialling of draft standards through field trial (only new Criterion to be tested)	NEC-SGP (5)	
6 Standard document revision and production	Format, produce and launch new standards	New edition of standards are produced and released to the sector	1/9/10 – 4/10/10	Ensure formatting of new standards is correct	Project Manager	NEC-SGP members, secretariat and key stakeholders present at launch
				New standards are launched during ASC 2010	Senior Project Officer	
				Online version is made available	Publications/ IT	
				Complimentary copies sent to key stakeholders	Secretariat	

Over 100 individuals substantively participated in the review process, with an additional 30 providing periodic input and feedback. Participants were drawn from general practice stakeholders, including: healthcare professional associations; primary healthcare services; accreditation agencies; government agencies; and public health organisations. Their expertise spanned the fields of: project management; standards development and writing; primary healthcare practice; quality and safety improvement methodologies; accreditation implementation and surveying; and research methodologies.

The review and development process was shaped by five factors. First, identifying and delivering upon the requirements of the RACGP, and stakeholders associated with the standards, was reported as necessary for the credibility of the product. Second, identifying and communicating resource and time restrictions to participants, and observers within the sector, was required to enable the review to be completed as expected. Managing expectations and employing an effective communication strategy reinforced a collaborative approach and facilitated broad stakeholder engagement with the review; these being the third and fourth issues identified as essential for a positive development process leading to the acceptance of revised standards. Finally, the review project had to deliver a well structured, clearly written, evidence based, high quality document that was consistent with previous editions. One significant improvement suggestion emerged from the evaluation: a majority of participants agreed that consideration could be given to altering the standards revision process to conduct periodic reviews and progressive updates.

## Discussion

This study provides the first case-study evidence about processes invoked for the development and revision of accreditation standards [2], and lays the foundation for further work in this area [3,4]. The research reveals that the revision of accreditation standards is a major undertaking requiring considerable resources and expertise, drawn from a broad range of stakeholders. Industry acceptance of the standards produced was found to be related to a collaborative, inclusive process, grounded by clinical evidence and process reviews, which promoted stakeholder participation. These findings support previously reported, non-empirical assessments, of how to approach the task [1]. For other accrediting bodies the study provides three things: insight into a difficult and challenging process; encouragement to investigate and make public their own experiences; and, a template and structure to follow to undertake such forensic examinations.

The project was completed through the combined efforts, or distributed leadership [19], of more than 100 people over a 12 month period. Key influences on the review process were: project requirements and stakeholder expectations; resource and time restrictions; collaborative team approach; stakeholder engagement; and the product required. The revision process necessitated the delicate balancing of these issues to maintain cohesion and continued participation between diverse and distributed stakeholders over an extended time period. Methodological rigour, as recognised by the six phases, was applied by the RACGP in the development, piloting and revision of materials. The commitment and effort of agency staff and committee members, who efficiently used resources with strict time constraints, enabled the efficient completion of the project. RACGP evaluation showed that stakeholder acceptance of the revision process and the revised standards produced was based on their perception of a transparent, inclusive and rigorous process implemented by the College [1].

## Conclusion

The revision of accreditation standards requires collaboration from a diverse range of professionals, with considerable resources and expertise. The collaborative, inclusive process employed engaged stakeholders and promoted the acceptance of the revised standards by the sector.
